# A Middle-Out Modeling Strategy to Extend a Colon Cancer Logical Model Improves Drug Synergy Predictions in Epithelial-Derived Cancer Cell Lines

**DOI:** 10.3389/fmolb.2020.502573

**Published:** 2020-10-09

**Authors:** Eirini Tsirvouli, Vasundra Touré, Barbara Niederdorfer, Miguel Vázquez, Åsmund Flobak, Martin Kuiper

**Affiliations:** ^1^Department of Biology, Norwegian University of Science and Technology, Trondheim, Norway; ^2^Department of Clinical and Molecular Medicine, Norwegian University of Science and Technology, Trondheim, Norway; ^3^The Cancer Clinic, St. Olav’s University Hospital, Trondheim, Norway

**Keywords:** logical model simulations, drug synergy prediction, systems medicine, model validation, middle-out modeling, model curation, cancer cell fate decisions

## Abstract

Cancer is a heterogeneous and complex disease and one of the leading causes of death worldwide. The high tumor heterogeneity between individuals affected by the same cancer type is accompanied by distinct molecular and phenotypic tumor profiles and variation in drug treatment response. *In silico* modeling of cancer as an aberrantly regulated system of interacting signaling molecules provides a basis to enhance our biological understanding of disease progression, and it offers the means to use computer simulations to test and optimize drug therapy designs on particular cancer types and subtypes. This sets the stage for precision medicine: the design of treatments tailored to individuals or groups of patients based on their tumor-specific molecular cancer profiles. Here, we show how a relatively large manually curated logical model can be efficiently enhanced further by including components highlighted by a multi-omics data analysis of data from Consensus Molecular Subtypes covering colorectal cancer. The model expansion was performed in a pathway-centric manner, following a partitioning of the model into functional subsystems, named modules. The resulting approach constitutes a middle-out modeling strategy enabling a data-driven expansion of a model from a generic and intermediate level of molecular detail to a model better covering relevant processes that are affected in specific cancer subtypes, comprising 183 biological entities and 603 interactions between them, partitioned in 25 functional modules of varying size and structure. We tested this model for its ability to correctly predict drug combination synergies, against a dataset of experimentally determined cell growth responses with 18 drugs in all combinations, on eight cancer cell lines. The results indicate that the extended model had an improved accuracy for drug synergy prediction for the majority of the experimentally tested cancer cell lines, although significant improvements of the model’s predictive performance are still needed. Our study demonstrates how a tumor-data driven middle-out approach toward refining a logical model of a biological system can further customize a computer model to represent specific cancer cell lines and provide a basis for identifying synergistic effects of drugs targeting specific regulatory proteins. This approach bridges between preclinical cancer model data and clinical patient data and may thereby ultimately be of help to develop patient-specific *in silico* models that can steer treatment decisions in the clinic.

## Introduction

Computational models that describe biological systems can help to provide insight into how these systems control regulatory events at the molecular level (Smolen et al., 2000; Le Novère, 2015). The ability to correctly predict the effects of systems perturbations by *in silico* simulations is a good indicator of how well the computational model represents biological reality. Indeed, computer models for diseased systems are being used to simulate drug perturbations and to develop, evaluate and prioritize putative drugs *in silico* (Flobak et al., 2015; Rubio-Perez et al., 2015). Approaches that use quantitative modeling rely on information including kinetic rate constants for regulatory components and their interactions, but this type of detailed quantitative data is only available for a small fraction of the regulatory interactions that underlie cell fate decision mechanisms. The much more abundant availability of binary molecular interactions, also defined as ‘causal statements’ (Touré et al., 2020) allows the use of the Boolean formalism as a powerful alternative mathematical framework for *in silico* simulation studies. The ability of Boolean models to represent discrete levels of a system furthermore complies well with the need for basic representations of cellular states, as these equate to stable states of regulatory networks that are interconnected through logical rules that may reach new stable states under different conditions, e.g., normal, diseased, and drug-perturbed. In systems medicine efforts to understand cancer, Boolean networks have been used previously to model biological systems driving cancer and were found useful for studying tumor progression and understand cancer signaling mechanisms (Srihari et al., 2014; Pirkl et al., 2016), predict tumor metastatic capabilities and therapy resistance (Srinivas, 2015), identify cancer-specific biomarkers, driver genes, drug targets (Irurzun-Arana et al., 2017; Sahoo et al., 2018; Qiu et al., 2019), and predict drug effects (Fumiã and Martins, 2013; Azuaje, 2017), including the possible synergistic effect of combinations of drugs (Flobak et al., 2015). Depending on the purpose of computational simulations, Boolean models can describe either a very specific process, such as a specific cancer-related signaling pathway (Grieco et al., 2013), or a collection of processes that together result in a biological phenomenon, such as the signaling pathways involved in metastasis. These models can vary in size, but they rarely comprise more than some tens of components.

In regulatory models based on the Boolean mathematical framework, a model component, also called ‘node,’ can either be active or inactive, which in Boolean algebra can be represented as 1 and 0, respectively. The state of a particular node (referred to as local state) is updated according to logical rules that capture the regulatory effects (activation or inhibition) of all the regulators of that node in the network, taking into account their activity state (Glass and Kauffman, 1973; Thomas, 1973). Logical rules in Boolean models follow the logical formalism and employ the operators AND, OR and NOT. Each Boolean model can be represented as a graph of nodes connected by a set of directed and signed edges, representing the causal interactions between the nodes. The same network graph can support multiple Boolean models, with logical rules specific for the system that the model should represent. Starting from an initial state, Boolean models that adequately represent biological systems are able to reach only a limited set of stable states (often only one), called attractors (Naldi et al., 2009; Helikar et al., 2012; Naldi et al., 2018), which can be considered as the mathematical equivalent of cellular states. Attractors can refer to a single stable state (singleton attractor), a set of stable states that repeat themselves in sequence (simple or complex cyclic attractor), or a set of stable states in which the system randomly oscillates (Wang et al., 2012; Irurzun-Arana et al., 2017). If a Boolean model can reach a stable state in which its node activities match experimentally observed activity states of their biological counterparts (e.g., the results of biomarker analysis), it indicates that the model captures to some extent relevant aspects of the biological system.

Boolean modeling toolkits (Gonzalez et al., 2006; Naldi et al., 2018) provide for a variety of analyses that can be further used to test, validate and enhance a model. Apart from being descriptive of a biological system and identifying attractors that comply with a particular state of a cell, Boolean models can also be predictive and be explored to simulate cellular behavior under perturbed conditions (Joo et al., 2018). Perturbation analysis allows the simulation of a system under different conditions, similar to knock-out, over-expression, or chemically induced perturbations in laboratory experiments. Such simulations can be designed for a variety of purposes, e.g., to analyze the regulatory system *per se* and identify critical nodes whose perturbation leads to significant functional changes in the system, thereby generating hypotheses as to their biological function in the system. Attractor analysis is also important to identify trajectories (a series of states that the network traverses through while reaching a stable state) in the system’s behavior (Huang et al., 2005). In the case of gene regulatory networks, attractors are usually associated with specific phenotypes (Cho et al., 2016; Yang J. M. et al., 2018). Furthermore, a disruption of the balance found in these stable states of normal cellular systems can many times be associated with specific diseases, including cancers, allowing the mechanistic understanding of cancer development and progression (Bachmann et al., 2012), which can provide an important advantage when designing cancer therapies.

This paper focuses on a specific use of Boolean modeling, namely its use for computer simulations to identify effective combinations of targeted drugs that act synergistically in growth inhibition of a set of cancer cell lines. It is well known that a combination of drugs can have a higher effect on treated cells than the individual drugs alone would suggest if their effect were only additive (Roell et al., 2017). This effect, called drug synergy, results from systems interactions between the drugs and may yield a higher treatment efficacy. The use of synergistic drug combinations may address some of the current treatment limitations in cancer by reducing the emergence of drug resistance, which is frequently observed with single-agent therapies (Al-Lazikani et al., 2012; Gottesman et al., 2016), and lowering the chance of potential side effects and toxicity because the individual drugs can be used at lower dosages (Crystal et al., 2014; Trairatphisan et al., 2016). The use of drug combinations may serve as a stepping stone toward precision medicine, in which limitations of single-agent treatment, such as low response rates and acquired drug resistance, may be overcome by treatment regimes that use drug combination therapy optimized for the individual patient (Madani Tonekaboni et al., 2018). Combinatorial treatment refers to the targeting of multiple molecular components of a tumor cell-fate decision network, either by the combination of two or more targeted drugs or by combining targeted drugs with other therapies like immunotherapy, antibody-based therapy, and chemotherapy, with the aim to exploit synthetic lethality and tumor vulnerabilities and dependencies to treat cancer (Al-Lazikani et al., 2012).

With the availability of a relatively large number of targeted drugs (Yu et al., 2019), this may provide for a substantial number of potentially powerful combinations of drugs, but despite the availability of automated screening platforms using efficient high throughput technologies, the testing of combinatorial drug effects in the laboratory depends on vast amounts of large-scale dose-response data that is extremely time and resource-demanding (Pirkl et al., 2016; Joshi and Durden, 2019). The collection of all possible combinations of the large repertoire of targeted drugs presents a vast search space, and the number of possible interactions that need to be screened quickly becomes unmanageable, especially when taking into account different drug doses, combinations with more than 2 drugs, timing effects of drug administration, and the high intratumor, interpatient and cancer type variability that needs to be replicated in assays. Consequently, the screening for potential synergy is currently conducted mainly on compounds with an already known effect and/or where the combination of specific drugs makes sense based on empirical observations, significantly limiting the subspace of possible combinations that are actually tested (Cheng et al., 2019). *In silico* methods, therefore, pose an attractive pre-screening possibility, provided that the computer predictions can reliably limit the experimental search space (Crystal et al., 2014; Tolcher et al., 2018). More specifically, this means that predictive models must accurately predict the cellular response to medication, reveal the potential synergy between different drugs, produce few or no false-negative predictions (potentially powerful drug combinations that would be excluded from further testing) and preferably also few false positives (drug combinations that in further testing prove to be ineffective). Computational models that meet these criteria can alleviate the screening burden and create insight in the molecular mechanisms that lead to perturbational synergy (Flobak et al., 2015; Jeon et al., 2018; Madani Tonekaboni et al., 2018; Cheng et al., 2019; Tang et al., 2019).

Therefore, it is of high importance to develop high-quality logical models for predicting drug synergies and validating them by testing against experimental observations. The construction of computational models of biological systems can either involve a top-down approach that uses genome-wide omics data analysis to reveal the underlying regulatory network structure, or a bottom-up approach, in which a regulatory network is built from single entities and their interactions, often based on literature that describes their detailed analysis in various experimental settings (Shahzad and Loor, 2012). Bottom-up approaches are usually based on the manual curation of models, focusing on entities of interest, such as biological entities that are also drug targets, or driver genes for cancer. During this manual curation, the modeler many times confronts a series of subjective decisions on the relevance of entities, interactions and, more generally, the specific cellular processes to incorporate in a model, to properly represent a biological system. For the purpose of assessing the effect of particular perturbations, there is the additional challenge to identify and encode multi-level nodes that can be directly associated with a phenotype and, thus, serve as phenotypic readouts in the model. These phenotypic output nodes provide a convenient way to assess and quantify the effect of the drugs *in silico* simulations.

Here it is presented how a top-down multi-omics data analysis can identify candidate genes that should be considered for addition to an existing model, serving as seed genes that provide guidance for additional bottom-up modeling. The cell signaling components used were highlighted by the analysis of multi-omics data from the Consensus Molecular Subtypes (CMSs) (Guinney et al., 2015) study of colorectal cancer (CRC), to expand the generic cancer cell fate decision network CASCADE 2.0 that was built previously by our group (Niederdorfer et al., 2020). This approach effectively constitutes a middle-out strategy that allowed the expansion in a pathway-centric manner, capturing processes that were highlighted as possibly important for CRC subtype development. Furthermore, the model was manually partitioned into functional subsystems, named modules, allowing a continuous switching between top-down (finding modules and seed genes) and bottom-up modeling (module completion) during the manual curation of each module, in order to comprehensively capture cell fate decision mechanisms. Additionally, modules served as a “binning principle” of nodes and regulatory relationships, providing an intermediate network level, placed between the individual binary interactions and a fully connected network. This allowed for a multilevel assessment of the system, focusing on the modular regulatory effect on output nodes, and their perturbational response to targeted drugs. The evaluation of the performance of the model in predicting experimentally validated synergies of combinations of 18 established cancer drugs in a panel of eight cancer cell lines revealed that the model performs similarly well for a majority of carcinoma cell lines in the panel, and not only for colorectal cancer that it was originally specialized for. Our results suggest that a middle-out modeling approach may be appropriate for optimizing the representation of specific cancers or cancer cell lines, or indeed other disease types for which multi-omics data is available.

## Materials and Methods

### Tools, Data Standards, and Exchange Formats

An overview of the software tools and their versions used in this study can be found in the Supplementary Material. Genes and proteins were represented with the standard identifier nomenclature for each entity type, namely HUGO Gene Nomenclature Committee (HGNC) symbols and UniProt IDs, respectively. Several files are available at are publicly available at https://github.com/druglogics/cascade, with information about the CASCADE 3.0 model: the model’s interactions as a Simple Interaction File (SIF); a file containing the supporting evidence for each of the interactions in the model; a file with information about node translation and module assignment; and a cytoscape.cys file of the network and its topology as shown in Figure 3. The github repository also contains information about other CASCADE models, including the CASCADE 2.0 model that was used as the basis for this work.

### Model Assembly by Manual Curation

Logical models are usually created manually by carefully screening the literature for evidence that supports the linking of components and their regulatory relationships in a Prior Knowledge Network (PKN) that represents a biological system. The CASCADE 2.0 model is a manually curated logical model, representative for the most prevalent cancer types (Niederdorfer et al., 2020). The CASCADE 2.0 model consists of 144 nodes and 366 interactions, including two output nodes called *Prosurvival* and *Antisurvival*. Each node was annotated with its HUGO gene symbol. In the case of several isoforms, a family-node representative of all isoforms was used. Family nodes are notated with a \_f in their name, while protein complexes and genes are notated with \_c and \_g, respectively.

Niederdorfer et al. (2020) describe several model versions, including a version in which the model topology was refined so that it better recapitulates the biological mechanisms of the analyzed cell lines. In the current analyses, the more generic cancer model was used. These different models were all constructed according to the following design principles: (1) include targets of specific drugs for which the effects should be simulated; (2) contain entities that are known to be involved in specific or more general oncogenic processes, and (3) contain specific nodes that will allow a read-out of the state of the cell fate (phenotype output nodes): actively dividing (*Prosurvival*) or growth-inhibited/apoptotic (*Antisurvival*). In this study, the CASCADE 2.0 model was taken as a basis for extending into a logical model that contains the major components and processes that can be identified as significantly perturbed in one or more of the colorectal cancer Consensus Molecular Subtype datasets (see below), which was named CASCADE 3.0.

### Identification of Affected Processes in Consensus Molecular Subtypes of Colorectal Cancer

An expression-based classification of the patients in the TCGA-COAD cohort was performed according to the Consensus Molecular Subtypes (CMS) classification for colorectal cancer (CRC), as described in the Supplementary Material. This patient classification aimed to identify commonalities and differences between the four subtypes at a genomic, transcriptional and functional (i.e., pathway) level. All the subsequent analyses were conducted separately for each CMS class of patients unless stated otherwise, and *p*-values were adjusted using the Benjamini–Hochberg method, to correct for the false discovery rate (FDR) across multiple tests (Benjamini and Hochberg, 1995).

The omics data used in the current project (i.e., mRNA expression, somatic copy number variation and mutation data) were publicly available data published as part of the TCGA-COAD project (Cancer Genome Atlas Network, 2012). Data from patients that were not classified into one of the CMS classes were not used in the analyses, while non-tumorous data from adjacent tissues of the classified patients were used when needed (further discussed in the following sections).

#### Differential Expression Analysis

Using the RNA-sequencing data of TCGA-COAD, a statistical analysis of differential expression was performed on the transcriptomes of the tumor samples using the edgeR RNA-Seq expression analysis package (Robinson et al., 2010). Data from the same patient, but originating from different vials, portions, analytes or aliquots, were averaged. RNAs with very low counts across all libraries (*fewer than 6–7 counts*) and genes that were expressed in only one sample were discarded, as they were deemed not significant. Since the high expression of some genes in a sample can lead to the under-sampling of the others, a normalization step to correct for differences in the library sizes was performed. The same filtering and normalization steps were performed in available normal tissue samples of TCGA. The differential expression analysis (DEA) was carried out against this collection of normal samples, for all the subtypes. Protein-coding genes with an FDR-adjusted *p*-value of less than 0.05 and a logarithmic fold change (logFC) greater than 2 or lower than −2 were deemed significantly differentially expressed.

#### Somatic Copy-Number Alterations (SCNV) Analysis

The GISTIC 2.0 tool (Mermel et al., 2011) in the GenePattern platform (Reich et al., 2006) was used to identify genomic regions that were significantly amplified or deleted across the different subtypes, based on the amplitude of the aberrations as well as their frequency of occurrence across the tumor samples. For this analysis, masked segment copy number variation data of TCGA-COAD were retrieved and used. In masked data, segments with probes known to contain germline mutations are removed allowing the identification of the cancer-associated, somatic copy number variation. The recurrently aberrant regions and their containing genes were identified with a threshold FDR < 0.01.

#### Recurrent Somatic Mutation Analysis

The MutSigCV tool (Lawrence et al., 2013) in the GenePattern platform (Reich et al., 2006) was used to identify recurrent mutations in the cancer genome of TCGA-COAD patients. The mutational profile of TCGA-COAD patients containing information on mutation type, category and its effect, was used to. Recurrent mutations are identified by calculating the probability of a non-silent mutation to have happened by chance compared to the background mutation rate estimated by silent mutations and other patient-specific and position-based confounding covariates. A threshold FDR < 0.05 was used.

#### Functional Analysis by Enrichment

Initially, genes that were found to be either differentially expressed, located in recurrently aberrant chromosomal regions or recurrently mutated were considered important for colorectal cancer cells. To further investigate the functional role of the affected genes in each subtype, independent enrichment analyses were performed against the Reactome (Fabregat et al., 2018), Kyoto Encyclopedia of Genes and Genomes (KEGG) (Kanehisa and Goto, 2000), and Atlas of Cancer Signaling Networks (ACSN) (Kuperstein et al., 2015) databases. For KEGG and Reactome databases the clusterProfiler package was used (Yu et al., 2012) while the ACSNmineR package (Deveau et al., 2016) was used for ACSN. Results with FDR-adjusted *p*-values lower than 0.05 were considered significant. In parallel, similar enrichment analyses were performed with the CASCADE 2.0 components (Niederdorfer et al., 2020), to identify the main pathways and processes represented in this model. A comparison of the results of these analyses revealed the processes that were affected in the CMS classes but not significantly represented in the topology of the CASCADE 2.0 model.

### The CASCADE 3.0 Model

#### Middle-Out Expansion of the Initial Model

The middle-out modeling process was characterized by a combination of a top-down and bottom-up approach. As already described, the first steps were governed by the genome-wide analysis of relevant omics data, a typical approach in top-down modeling where correlations between genes or proteins are investigated by deploying various statistical and bioinformatics analyses. More specifically, the top-down step and overrepresentation analysis highlighted the affected processes in the different CMSs, after which the nodes of CASCADE 2.0 network could be annotated and mapped to these overrepresented signaling pathways and biological processes. However, as additional missing process and pathway components were added during the construction of CASCADE 3.0, the module assignment for some nodes had to be further refined, in ways that it better represented the role of these entities in the modeled system.

When most of the nodes were assigned to modules, four of the initial modules were divided into two segments: one containing entities involved in the core signaling pathway and the other containing the negative regulators of that pathway. The core signaling pathway included proteins involved in signal transduction, starting mostly from receptors sensing a signal and all the signaling proteins (i.e., the positive regulators of the response and the main effector of the pathway) that enable the regulatory response to the signal. The negative regulators were placed in the other module, including entities involved in negatively regulating the pathway’s main effector, meaning that they are involved in potential regulatory feedback loops, as seen for example in the WNT and MAPKs modules.

As a next step, a bottom-up approach was employed to expand the modules so that they comprehensively represent relevant pathways. As is common practice in bottom-up approaches, this step was focused on individual biological entities and their interactions, using a variety of databases, knowledge bases and sometimes literature. The expansion of the modules and the construction of the extended CASCADE 3.0 model was an iterative process of manual curation: Each module was manually checked against existing knowledge to comprehensively capture its intra- and inter-modular regulatory, causal interactions that would likely contribute to the overall cell fate decision mechanism that the model should represent. A detailed list of all the knowledge resources used during the curation processes is presented in the Supplementary Material. Most of the initial curation work drew on the cell signaling pathway database Signor (Perfetto et al., 2016), which, in combination with the primary modules from the original CASCADE 2.0, guided the addition of new nodes in each of the pre-existing modules. An important part of this curation process was the identification of the context under which an interaction was observed. In order to retain high confidence in the accuracy with which the model describes the biology of colorectal cancer cells, only regulatory interactions relevant for cells of tissues from which CRC subtypes originate were selected. In case interactions were reported in other tissues, additional literature was checked to decide whether to include or discard the interaction. Furthermore, interactions that were reported for specific biological processes not relevant to the biological system that the models should capture, for example, cardiac development, were omitted.

Taking into account the possible cross-talk of signaling pathways and the multi-functionality of many biological molecules, all nodes were examined for their potential participation in several pathways. Because of this, some nodes, including entities such as adaptor proteins or cytoplasmic kinases, were functionally attributed to multiple modules, but are presented and analyzed in CASCADE 3.0 only as members of one main module. The assignment to these modules was based on the available knowledge on the functional role of the nodes and the number of interactions it shared with the other members of that module.

#### Logical Modeling

The transformation of the expanded PKN into a Boolean model was done by the definition of logical rules that describe the overall regulatory input that each node receives: for this, the regulatory effect of each of the input nodes (activating or inhibiting) was combined with the logical AND, OR and NOT operators. The local state of each entity depends on the state of the combined set of nodes that regulate it. As described above, those regulations are captured in the logical rules that govern the update of the state of each node. As a point of departure, general logical rules that assume that an entity is active if *any* of its activators is active and *none* of its inhibitors is active were defined. According to the general rules, a protein’s activity will be downregulated by any active inhibitor, regardless of the upregulation input of one or more activators (Shmulevich et al., 2002).

According to the notation of the logical formalism, the rules of the nodes’ activities are of the form:

Node X = (Activator A OR Activator B … Activator n) AND NOT (Inhibitor A OR Inhibitor B OR … Inhibitor n)

Two additional “phenotype” nodes were added to the model, representing the two cellular states *Prosurvival* and *Antisurvival.* These nodes were implemented as multivalued nodes (with possible local states 0, 1, 2, or 3) which serve as cellular state readout and allow to assess the overall proliferative state of the system. The global state of the system is computed as the overall sum of the negative value of *Antisurvival* and the positive value of *Prosurvival*, ranging between −3 and +3.

### Drug Synergy Prediction

Drug synergy predictions were performed with a custom-built modeling pipeline that combines several software modules that together provide a highly automated computational framework for logical model assemble and simulations (Flobak et al., manuscript in preparation)^1^. The pipeline can customize a general logical model to a specific cell line, after which it uses a collection of models (ensemble) each equally fit to represent a cell type to predict the effect of a drug perturbation, as well as their potential synergies in case of drug combinations. Initially, omics data of a specific cell type are translated into entity steady state activities (1 or 0). Such omics data can be either genome-wide or biomarker-specific, and can include among others proteomics, genomics, and transcriptomics data, either separately or in combination. The omics data serves to produce a training set of steady state activities that the network nodes should display when the logical model reaches a stable state. As this is dependent on the exact configuration of the overall logical rules of the model, these logical rules are optimized with the help of a genetic algorithm that changes sets of logical rules and analyses the steady state values from the altered model against this training set.

The genetic algorithm iteratively “mutates” the logical rules of some nodes each time, by randomly switching between AND and NOT and then the global stable states of the mutated models are calculated using the BNReduction tool (Veliz-Cuba et al., 2014). The mutated models that show the highest fitness (their stable state node activities better resemble the data in the training set) are further mutated for a selected number of iterations.

This optimization process results in an ensemble of models, all having the same topology but with slight differences in their logical rule structures, each model of an ensemble complying more or less equally well with the regulatory system that should be represented. The logical model ensemble is next systematically subjected to a set of *in silico* drug perturbations by assessing the combinatorial effects of drugs on the models as observed by the combination of states of the phenotype output nodes. These output nodes are multi-valued (global state ranging from −3 to +3) and the state of these nodes is defined by the predicted local states of key nodes that provide ‘regulatory input’ to these phenotype nodes, such as the cyclins, MYC and other survival factors that add additively to Prosurvival and the caspases and other pro-apoptotic entities that add to Antisurvival. With the global state ranging from −3 (only activity from anti-survival nodes) to +3 (only activity from pro-survival nodes), the quantification of the effect of the drugs to the viability of a system after single and combinatorial drug simulations was possible. For example, a drug that results in a global state of −3 has a more prominent effect than a drug that results in a global state of −2 or −1. The global state of the combinatorial treatment was then compared to the global states of the treatment of each individual drug. If the drugs that together result in a viability (i.e., output nodes’ state) smaller than the minimum of the viability of each individual drug, they were scored as synergistic. These predictions were then compared to observed synergies validated by experimental data produced by the combination of 19 small molecule inhibitors and their 171 combinations (Flobak et al., 2019). As discussed in Niederdorfer et al. (2020), an inhibitor of PTEN (SF-1670) that was found to be under characterized regarding its off-target effects and was involved in the majority of synergies was not included in the analysis, reducing the data used to 18 small molecule inhibitors and their 153 combinations. The inhibitors were targeting various modules of the models and were tested in all eight cell lines used in the simulations. Furthermore, all drugs used in the screen were subjected to in depth characterization including an extensive target profiling, in associating the drugs and their targets with the model’s nodes. The overall performance of the model with respect to true positive, false positive, true negative and false negative drug synergy predictions was assessed using AUC-ROC curves as performance measurement (Sammut and Webb, 2017).

In this project, three different sets of inferred entity states were used as training data to the genetic algorithm. Two of the data sets include activity states inferred from two distinct sets of omics data, using the Paradigm tool (Vaske et al., 2010), while the third set contains protein activities inferred from transcription factor activity information, using the Viper tool (Alvarez et al., 2016). The first set of activity states from Paradigm, referred as *Combination-based*, makes use of cell line specific copy number variation, gene expression, RPPA for total protein abundance and RPPA for phosphosites to infer entity states. The second set of states from Paradigm, referred as *mRNA expression-based*, uses only the cell-line specific mRNA expression data. For the *TF activity based*, data from “Genomics of Drug Sensitivity in Cancer” (GDSC) project were used as an input for Viper.

The model optimization work indicated that larger sized training sets not necessarily correlated with higher AUC values. This might be explained by imperfections in the training data: inferred data, so not experimentally confirmed data, may have errors in it that limit the “freedom” available to the genetic algorithm to adequately fit model steady states to the real biological state of the system. For that reason, the *Combination-based* and *mRNA expression-based* datasets were reduced to only those nodes for which the smaller data sets also contained a predicted state. That also allowed a more direct assessment of training data sets with respect to their ability to correctly serve as local states that would be observed in biological reality. More details about the reduction of the training data can be found in the Supplementary Material. All three training sets were subsequently used to evaluate how the model performs for eight cancer cell lines (see Table 1).

**TABLE 1:** Description of the eight cancer cell lines used in the synergy prediction analysis.

Cell line	ID	Tissue	Disease
AGS	RRID:CVCL_0139	Stomach	Gastric adenocarcinoma
Colo205	RRID:CVCL_0218	Colon; derived from metastatic site: ascites	Colon adenocarcinoma
DU145	RRID:CVCL_0105	Prostate; derived from metastatic site: brain	Prostate carcinoma
SW620	RRID:CVCL_0547	Colon; derived from metastatic site: lymph node	Colon adenocarcinoma
MDA-MB-468	RRID:CVCL_0419	Breast; derived from metastatic site: pleural effusion	Breast adenocarcinoma
A498	RRID:CVCL_1056	Kidney	Renal cell carcinoma
SF295	RRID:CVCL_1690	Brain	Glioblastoma
UACC62	RRID:CVCL_1780	Skin	Melanoma

## Results

### Identification of Affected Processes

#### Omics Data Analysis

The candidates for regulatory network inclusion were identified through a multi-omics data analysis that included transcriptomic (i.e., gene expression data) and genomic (i.e., somatic mutations and copy number alterations) profiles of CRC patients, effectively identifying affected processes and pathways in these patients’ tumors. The differential gene expression analysis identified the highest number of affected genes and displayed a significant overlap between the differentially expressed genes in the subtypes, all involved in fundamentally dysregulated processes in cancer, such as DNA repair, cell adhesion, and cell cycle control. The identification of somatic copy number alterations (SCNAs) corroborated the profiles of the molecular subtypes and revealed both known and novel aberrant chromosomal regions. CMS2 and CMS4 displayed the highest number of SCNAs, whereas the two remaining subtypes had a low number of aberrant regions. Interestingly, a much higher number of genes was found correlating with deleted peaks than with amplified peaks, for all the subtypes. Among the 114 unique aberrant regions across all subtypes, five regions were altered in all subtypes. Four of those regions (16p13.2, 20p12.1, 5q12.1, and 4q22.1) showed deletions, while 8p11.21 was amplified in all the subtypes. The 20p12.1 region has been previously reported as recurrent in CRC (Davison et al., 2005), but there are no reports for the presence of known cancer genes in any of the regions. Some of the identified SCNAs have been previously reported for their involvement in other cancer types, but not in CRC. A number of genes located in these chromosomal regions have been associated with clinical characteristics of cancer patients and could potentially be investigated as biomarkers or drug targets for CRC (Coppedè et al., 2014). The somatic mutation analysis did not show any association between the mutation of specific pathways and specific subtypes, as the major signaling pathways known to be altered in CRC tend to be mutated in all the subtypes. Given its Microsatellite Instability (MSI) status resulting from a defective DNA mismatch repair machinery, CMS1 patients are expected to have a predisposition to hypermutability (Yu et al., 2019). For that reason, CMS1 patients had the highest number of recurrently mutated genes.

**FIGURE 1:**
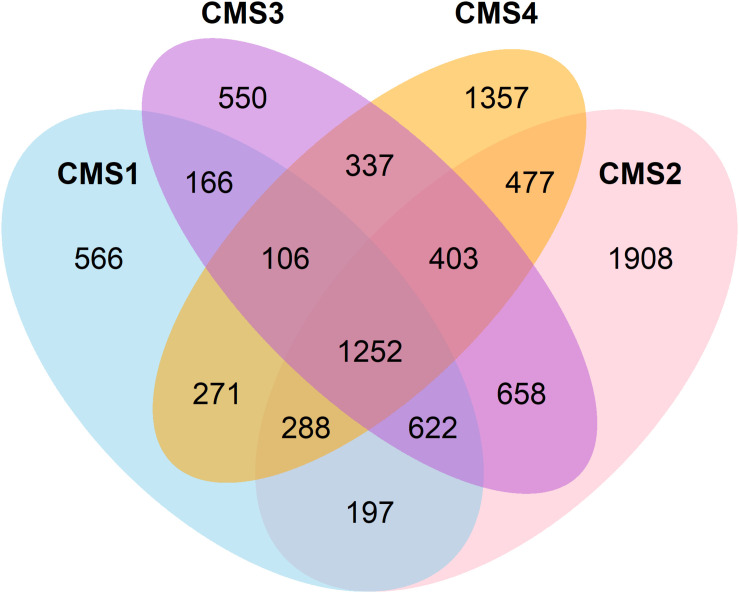
Venn diagram showing the overlap between genes affected in the four CSMs.

A list of the affected genes was produced for each subtype and classified as either differentially expressed in comparison to normal tissue, recurrently mutated, or located in a recurrently amplified or deleted region with respect to normal copy number. The total number of affected genes per category in each subtype is presented in Table 2, and their overlap in Figure 1.

**TABLE 2:** Total number of genes that were found to be affected in the omics data analysis.

Subtype	Recurrently mutated genes	Amplified genes	Deleted genes	Upregulated genes	Downregulated genes
CMS1	55	541	135	1625	1658
CMS2	6	438	2508	1793	1789
CMS3	11	12	2054	1185	1501
CMS4	10	587	3461	2003	919

**Affected genes are defined as genes that were either differentially expressed in comparison to normal tissue, amplified or deleted with respect to normal copy number or recurrently mutated*.*

#### Functional Analysis of CMS Genes by Enrichment

**FIGURE 2:**
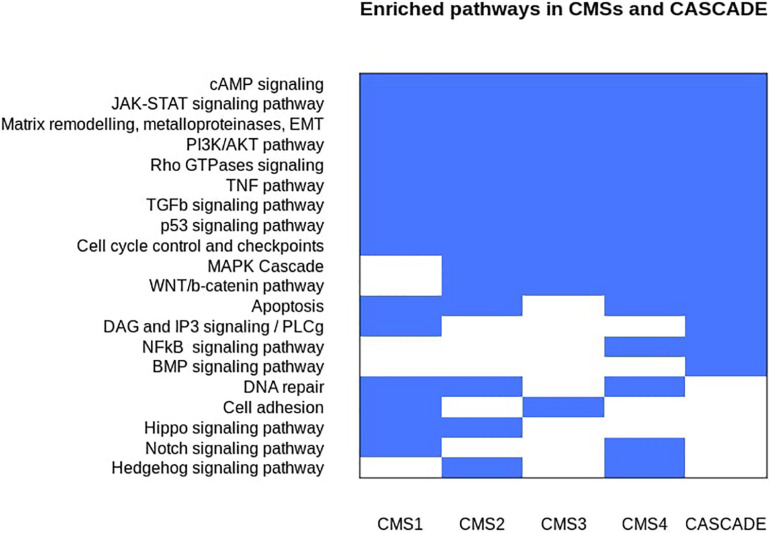
Discrete heatmap of the presence or absence of signaling pathways and biological processes that were identified as affected in the Consensus Molecular Subtypes of colorectal cancer and/or represented in the CASCADE 2.0 model. Blue colored cells represent the enrichment of a process in a subtype and/or the model.

**FIGURE 3:**
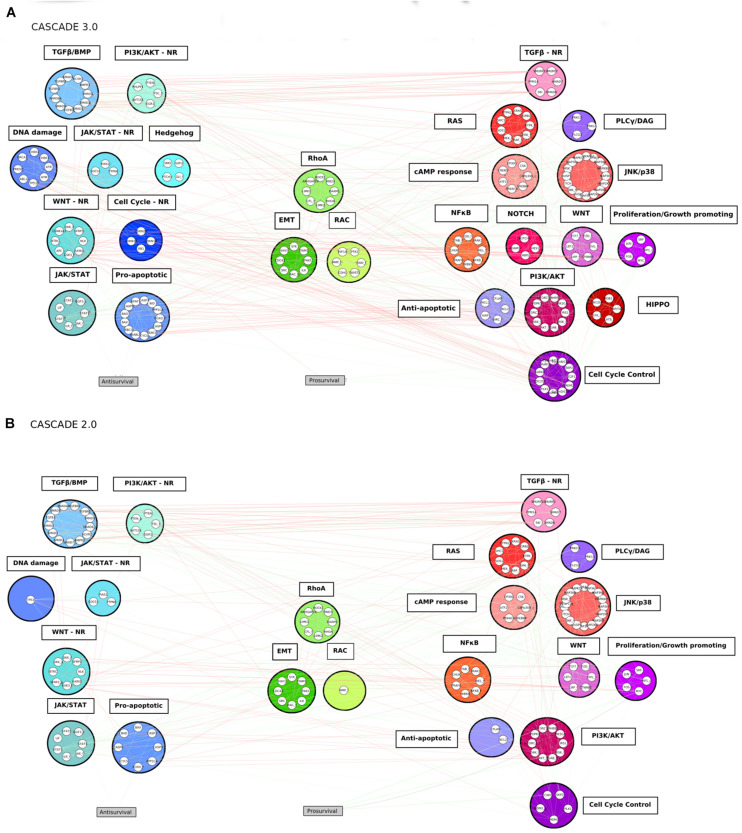
The CASCADE 3.0 **(A)** and CASCADE 2.0 **(B)** models. The nodes are grouped according to pathway modules. The modules are grouped based on their promotion of apoptosis (blue colored modules), metastasis (green colored modules) or proliferation (red colored modules) when the pathways they represent are active. The position of a module in the figure displays its proximity to the output node: the smaller the average shortest path of the module to the output node it is related to, the closer to the output it is placed. Empty slots in the CASCADE 2.0 topology show the individual nodes or complete pathway modules that were added in the CASCADE 3.0 model. Gray rectangular nodes represent the output nodes.

An analysis of the affected genes for their functional enrichment was performed against the ACSN, Reactome, and KEGG databases (Kanehisa and Goto, 2000; Kuperstein et al., 2015; Fabregat et al., 2018). The results are shown in Figure 2 and are represented as individual, non-redundant, cancer-related pathways. Non-cancer related pathways and processes mainly found in cancer-associated cells in the tumor microenvironment, such as cancer-associated fibroblasts and immune cells, were not included in the results as the model does not account for inter-cellular interaction. Since the model aims to represent regulatory interactions involved in signaling pathways, metabolic pathways that were found to be deregulated, especially in the case of the metabolic subtype, could not be represented in the model and thus were also excluded from the results. A similar enrichment and aggregation analysis was done for the nodes of the CASCADE 2.0 model and a comparison with the affected CMS pathways (see Figure 3) highlights the signaling pathways that are affected in CRC but were not included in CASCADE 2.0. The identified missing processes included the Hippo, Hedgehog, and Notch signaling pathways, as well as DNA repair and cell adhesion, all with well documented involvement in both CRC and cancer in general (Wierzbicki and Rybarczyk, 2015; Vinson et al., 2016; Wu et al., 2017; Boesch et al., 2018; Mirza-Aghazadeh-Attari et al., 2018).

### Construction of the CASCADE 3.0 Model

#### Manual Extension of the CASCADE 2.0 Model

Guided by the results of the top-down analysis and founded on prior knowledge from several databases and the literature, the CASCADE 3.0 model was constructed through the addition of nodes and edges to describe fundamentally dysregulated processes in all CRC subtypes. Those processes involved cell fate controlling processes such as cell cycle progression, regulation of apoptosis and response to DNA damage, as well as signaling pathways that were identified to be missing from the CASCADE 2.0. The final network consists of 183 nodes and 605 edges (see Figure 3). In addition to the inclusion of new nodes and interactions, small refinements were performed in the model. Nodes representing genes (*notated with _g in CASCADE 2.0*) were removed from the model, and replaced with their gene product node, including their regulatory interactions. Additionally, the CK1_f node, containing CSNK1A1, CSNK1D, and CSNK1E isoforms was split into two nodes, due to the involvement of the two latter isoforms in a newly added Hippo pathway module. Finally, in order to more accurately represent the regulation of the cell cycle by MYC (Bretones et al., 2015), the edge representing the direct interaction of MYC with the *Prosurvival* output node was replaced by an edge representing the promotion of proliferation through the activation of CCND1. Finally, in addition to the *Prosurvival* and *Antisurvival* output nodes, a new output node representing *Metastasis* could be included, based on the observation that several pathways were involved in metastasis-related processes (e.g., Epithelial-to-Mesenchymal transition and cell motility). However, due to the lack of appropriate screening data for this effect, it was omitted from the model, but it could be considered in future extensions of the model.

#### Topological Comparison of Original and Extended Models - Final Modules

The 183 nodes of the extended model were grouped into 25 manually curated pathways modules representing altered pathways or functions in the CMSs of colorectal cancer. Additionally, four of those modules (WNT, PI3K/AKT, TGFβ, and JAK/STAT) represent the negative regulators of a specific pathway and its respective main effector. An example of such a set of negative regulators is the β-catenin destruction complex. The components of the complex (i.e., APC, AXIN1, CK1, and GSK3B), are involved in the WNT pathway, but they negatively regulate its main effector (β-catenin), so they were assigned to a separate module (WNT negative regulators module) than the core signaling pathway (WNT module). The resulting modules vary in size and structure, and nodes grouped in a module do not necessarily share interactions with each other. This is specifically the case for modules with entities exerting similar regulatory functions (e.g., the Anti-apoptotic module), but do not necessarily interact with each other to achieve that function. The modules share numerous interactions with each other, a reflection of the fact that biological pathways cannot be delineated as completely independent groups, and perturbations in one module are likely to affect the behavior of other modules. In biological systems, module cross-talk can give rise to emerging functions that differ from their original functions (Lorenz et al., 2011). This is especially true when cells execute more complex behaviors, such as invasion in cancer systems, which are often controlled by many processes and a result of the interaction of many modules (Koutsogiannouli et al., 2013).

A side-by-side comparison of the topologies of the two networks is shown in Figure 3, allowing an easy identification of the added or expanded modules in CASCADE 3.0. Of the 144 nodes of the CASCADE 2.0, 36 were among the genes affected in at least one of the molecular subtypes of CRC and these were assigned to 16 different modules. These modules represented key oncogenic processes, such as proliferation-promoting transcription factors, apoptosis, the JAK/STAT signaling pathway, and MAPK cascades. The majority of the affected genes present in CASCADE 2.0 was found to be part of signaling pathways whose dysregulation is considered to be a driver event in colorectal cancer: the PI3K/AKT, WNT and T ransforming Growth Factor-β signaling pathways (Tiwari et al., 2018). While only five modules representing missing pathways identified in the enrichment analysis step were added, all other modules were expanded either with a few entities or, in some cases, a substantial number of them. The modules that had the most nodes added represented processes such as adhesion and EMT, negative regulation of apoptosis, cell cycle control and checkpoints, and DNA damage response and repair.

### Drug Synergy Prediction

#### Evaluation of the Model’s Overall Performance

The drug synergy predictions were performed with three sets of models, each trained with a different set of inferred node activity states: TF-, Combination-, and mRNA expression-based. Model optimization and drug synergy scoring was as described in Methods, and prediction performance was benchmarked against experimental data obtained for eight cancer cell lines (Flobak et al., 2019) and evaluated using AUC values that define the ability of a model to distinguish experimentally validated synergies and non-synergies (Sammut and Webb, 2017).

**FIGURE 4:**
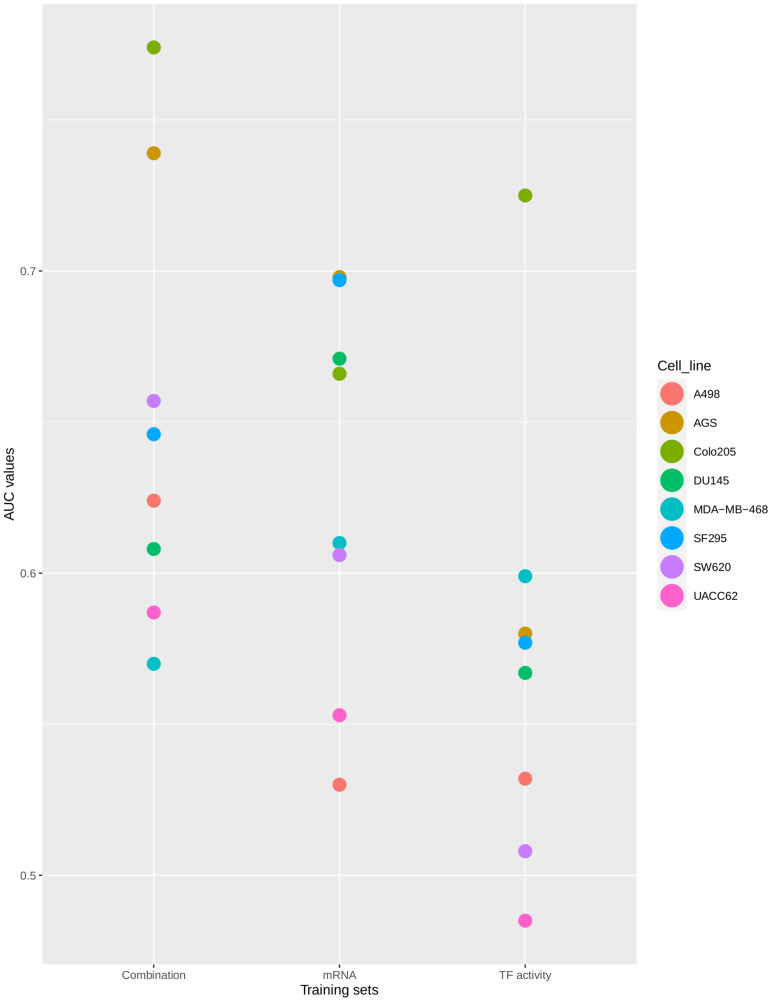
Synergy prediction AUCs per cell line. The plot shows the AUC values of the ROC curves produced for drug synergy prediction performance using CASCADE 3.0 optimized to three different training sets. Colors represent different cell lines. The AUC values are plotted on the *X*-axis. The training sets are shown on the *Y*-axis.

The distribution of AUC values between 0.5 (no prediction efficiency) and 1.0 (optimal model predictions) shows that the model’s performance depends both on the training data that was used and the cell line for which predictions were produced. As shown in Figure 4, models tend to perform better when trained to the *Combination-based* training set, and model performance can be very high for some cell lines, while for other cell lines drug synergies prove to be difficult to predict with any training set. The model displayed a (relatively) good performance for both the colorectal adenocarcinoma (Colo205) and gastric adenocarcinoma (AGS) cell lines. Synergy predictions for the prostate carcinoma cell line DU145 were consistently of moderate accuracy (AUC values ∼0.6–0.7), while predictions for the melanoma cell line (UACC62) was consistently the poorest, with an AUC value lower than 0.5 for the *TF activity* training data set. Prediction performance for the other cell lines range from moderate to very high, depending on the training set used. In order to ensure that the performance of the *Combination-based* training set was significantly improved when compared to the other training data sets, a one-sided t-test was performed. The comparison of the performance between the *Combination-* and *TF activity-based* training data showed significant improvement (*p*-value = 0.024). At the same time, the difference between the Combination- and mRNA-based training data sets was not significant (*p*-value of 0.2671). However, all but two cell lines have an improved performance with the Combination-based data and these data were therefore selected as the one yielding the highest performance. This variance in performance could indicate the inability of specific computational tools to correctly infer node activity states for specific cell lines, the importance of these states when training the model, or that models for these cell lines need specific topology optimization in addition to the logical rule optimization, to make them more stable with respect to the training data.

**FIGURE 5:**
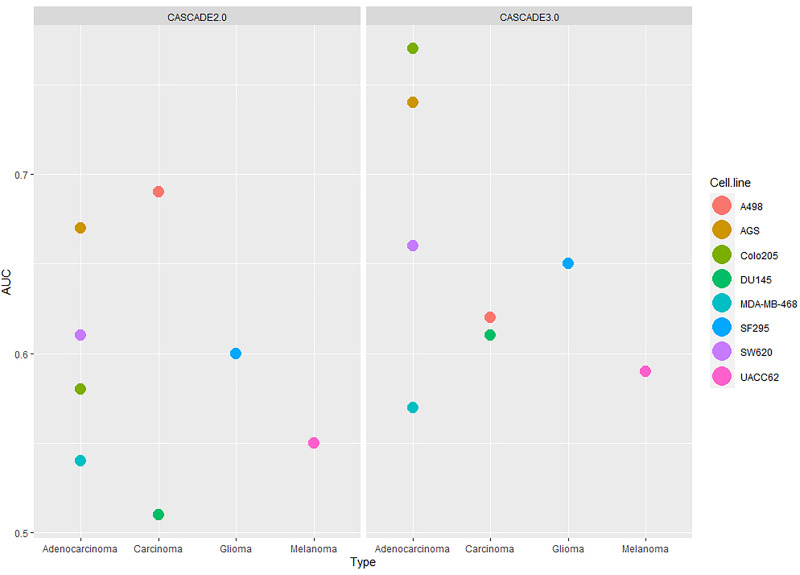
Comparison of the performances of CASCADE 2.0 **(left)** and CASCADE 3.0 **(right)** with respect to their ability to predict drug synergies. Performance is measured in AUC values and presented in the *y*-axis. On the *x*-axis, cell lines are grouped based on their tissue of origin and each point of the plot represents a different cell line.

As the *Combination-based* training data set was the most informative one, this set was used as the basis for a comparison of the performance between the initial (i.e., CASCADE 2.0) and the updated (CASCADE 3.0) model. The obtained AUC scores for each cell line are shown in Figure 5, with the model performances shown side by side. The statistical significance of the difference between the performances of the two models was computed by a paired Wilcoxon signed-rank test. The median AUC of CASCADE 2.0 was found to be significantly less than the median AUC of CASCADE 3.0 (*p*-value of 0.03). As seen in Figure 5, with CASCADE 3.0, there was a considerable improvement of the performance in all cell lines except the kidney carcinoma cell line, A498. As mentioned above, CASCADE 3.0’s improvement was most conspicuous for the Colo205 cell line. While CASCADE 3.0 overall seems to perform better for almost all cell lines, the range of improvement is most noticeable for cell lines originating from adenocarcinomas. This may indicate that the model extensions may better capture processes relevant to adenocarcinomas in general, rather than those specific for colorectal cancer and its subtypes.

#### Analysis of the Individual Predicted Synergies in Different Cell Lines

The mapping of the interactions between the 18 drugs used in this project and their target-entities revealed that the 20 entities of the model that serve as a target to those drugs are members of only 11 of the 25 modules. Two of the 18 drugs had no experimentally observed involvement in any synergy, reducing the number of modules involved in drug synergies to ten. Multiple drugs included in the screening and simulations were found targeting entities belonging to the same module. Specifically, four, three and two different drugs were targeting the PI3K/AKT, JNK/p38, and JAK/STAT modules, respectively. As many times cancer therapies take advantage of the dependency of cancer cells on an oncogene and/or loss of a tumor suppressor, and with the aforementioned pathways being among the most frequently altered pathways in several types of cancer, it is expected that multiple drugs have been designed to target these specific pathways (Thomas et al., 2015; Mayer and Arteaga, 2016; Martínez-Limón et al., 2020). The remaining seven modules included only one drug target each.

**FIGURE 6:**
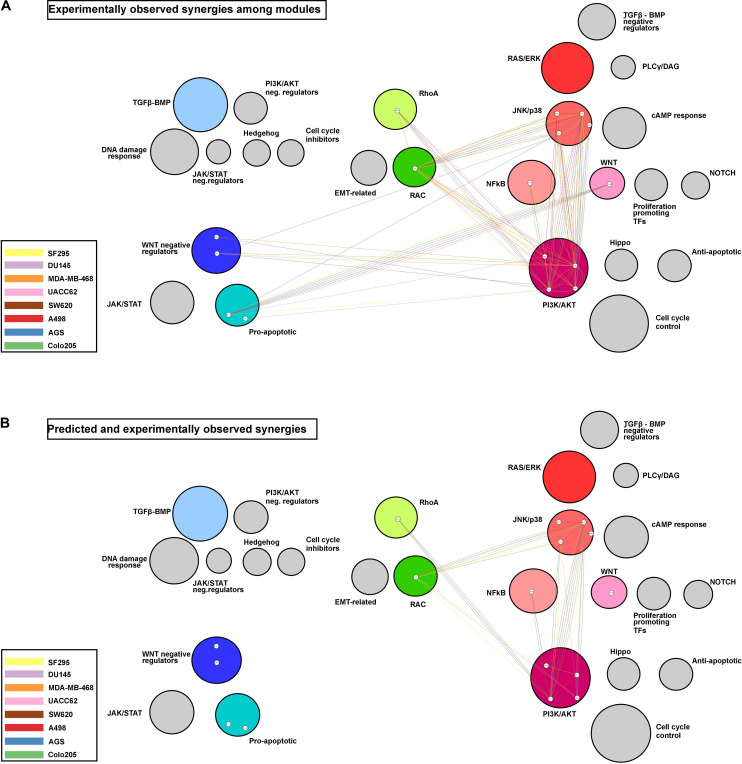
Network modules and synergy prediction. Network representing the experimentally observed **(A)** and the predicted and experimentally observed **(B)** synergies as edges connecting the drug targets involved. Colored circles represent modules with targeted nodes, while circle size reflects the number of nodes in each module. The colors of the edges represent the cell line for which a synergy was observed and/or predicted.

To visualize potential patterns in the ability of the model to correctly predict experimentally observed synergies, Figure 6 displays the synergies in a module, represented as connecting edges between the drug targets, and in a cell line-specific manner. The Figure 6A shows all experimentally observed synergies, and Figure 6B shows the observed synergies that were also predicted. Only predictions obtained with the best performing training data set (*Combination-based*) are shown.

Three modules, PI3K/AKT, JNK/p38, and RAC, appear to be involved in the majority of the observed synergies, with most of the synergies observed in at least four cell lines. Additionally, the PI3K/AKT and JNK/p38 modules presented cases of intra-module synergies, with targeting of two entities in these individual modules resulting in synergistic response. Some modules, such as the one composed of the WNT negative regulators, were involved in synergies observed in only a few cell lines. It is interesting to note that the model fails to correctly predict drug synergies for the two modules with drug targets displayed toward the left in Figure 6 (i.e., the modules that promote apoptosis when active), for any of the cell lines.

## Discussion

As standard treatment plans for cancer patients are often thwarted by acquired drug resistance of tumors, or the adverse effects and toxicities of monoregimen therapies (DeVita et al., 1975) combinatorial treatment with multiple chemical agents is being proposed as a solution (Kummar et al., 2010; Al-Lazikani et al., 2012; Madani Tonekaboni et al., 2018; Goldman et al., 2019). *In silico* screening of drug combinations may be particularly helpful in the pre-clinical stage, as it may serve to identify large numbers of combinations that need not be tested because they are unlikely to exhibit synergy (Celebi et al., 2019). *In silico* pre-screening may therefore solve many of the logistical and financial challenges that testing the enormous combinatorial drug compound space poses for screening facilities, provided that the computations predictions are of sufficient quality. Since the testing in the laboratory will only include a subset of the possible combinations (Cheng et al., 2019), it is critical to have a pre-screening procedure that produces low numbers of false negatives, as any potential blockbuster combination among them would not be tested.

Several approaches for *in silico* identification of drug synergies have been explored, some of them employing the computational modeling framework (Klinger et al., 2013; Miller et al., 2013; Flobak et al., 2015; Vitali et al., 2016; Eduati et al., 2017; Niederdorfer et al., 2020), as the current paper. In addition, machine learning (Jeon et al., 2018; Preuer et al., 2018; Sidorov et al., 2019; Yang et al., 2020), graph theory (Li et al., 2018), and multi-omics integration and analysis (Celebi et al., 2019; John et al., 2020) have been used. While machine learning approaches can be both highly flexible and accurate, there are certain limitations that should be acknowledged, such as the need to include expensive, hard to obtain training datasets, and for certain approaches (e.g., neural networks) they offer limited insights to what features confer predictability. On the other side, with logical models that make use of the abundantly available interaction data, synergy predictions can be successfully obtained from combining a prior knowledge network with observations, without the need for actual drug synergy training data, as demonstrated in the current manuscript. However, in a community effort to assess the computational prediction approaches (Menden et al., 2019), it was underlined that *in silico* synergy prediction remains a challenge even with using training data, and before such applications reach the clinic certain obstacles have to be overcome. One of these, the ability to tailor a computer model to the unique patient-specific molecular profiles, is key for the development of personalized therapies (Menden et al., 2019). To overcome this bottleneck, several methods to integrate patient-specific molecular characteristics have been proposed, with most of them exploring the use of multi-omics and perturbation data. As also demonstrated by our paper as well as others, logical models can be trained to both omics (Silverbush et al., 2017; Béal et al., 2019) and/or perturbation data (Fey et al., 2015; Eduati et al., 2020) in order to be further specified to specific cell lines or even patients.

The main aim of the project was to explore the use of multi-omics data to further extend and enhance a logical model that was produced by a manual curation effort. Analysis of colorectal tumor-derived omics data was used to define pathway modules representing functionally-related groups of proteins. Modules relevant for colorectal cancer were obtained through a workflow that combined multiple omics data to identify pathways and processes affected in the consensus molecular subtypes (CMS) of colorectal cancer (CRC). This top-down approach efficiently revealed CRC-specific processes, all with well-documented roles either in general tumor formation, or specific colorectal and/or general adenocarcinoma tumor ontogenesis. Next, a bottom-up approach was performed to extend an existing cancer model (CASCADE 2.0) with the new network nodes together with additional functionally relevant pathway and module components, to produce CASCADE 3.0. These approaches together exemplify an efficient middle-out workflow for cell fate decision network building, combining the best of well established top-down and bottom-up modeling approaches (Xavier et al., 2014). The top level results (affected pathways) were used to set the boundaries regarding the processes that should be present in the model, while the study of the individual entities involved in these pathways (bottom level) was guiding the curation and integration of these entities and their interacting partners in the system. The main advantage of this approach is that it provides a direct link between a collection of clinically relevant molecular phenotypes for very specific cancer (colorectal cancer subtypes) and a general model scaffold for cancer-related cell fate decisions. More specifically, it provides a modeler with very direct guidance for model refinement, essentially a blueprint of the modules whose inclusion should be considered. Similar workflows should allow model refinements for essentially any cellular system, provided that ample genome-wide information of that biological system is available. The modeler, however, will still face the responsibility to critically assess each model extension and guarantee the overall quality of the final model.

The broad availability of curated pathway resources and the definition of condition- and context specific modules could alleviate this workload, but it would be even better if a collection of reusable and interchangeable modular structures would be available that could be added or removed according to the different modeling purposes for different biological systems. The capacity of modules as building blocks has indeed been investigated in various types of biological networks (Segal et al., 2004; Schroeder, 2015), and the interest in building models in a modular manner is increasing.

To assess the quality of the CASCADE 3.0 model to predict drug synergies, simulations were performed for eight different cancer cell lines from various tissue origins, using three training sets for model configuration. The expectation was that the CMS extensions to the CASCADE 2.0 model would enhance the model performance for colorectal cancer. Model predictions were tested against experimentally observed synergies, and the AUC values indicated that CASCADE 3.0 had an improved prediction for Colo205, a colorectal adenocarcinoma cell line. However, the second adenocarcinoma cell line, SW620, displayed a more variable performance across the training data, with AUC values ranging from almost random (∼0.5) to 0.7. Interestingly, a multi-omics analysis of 34 colorectal adenocarcinoma cell lines (Berg et al., 2017) classified Colo205 and SW620 to different colorectal consensus molecular subtypes, as they have significant molecular differences. Among others, their CNV and gene expression profiles are quite distinct, causing Colo205 to be classified as a colon-like cell line, and SW620 as an undifferentiated cell line. These molecular differences and different subgroup classifications may indicate different underlying cellular signaling network activities or even different network topologies of these seemingly similar colorectal cancer cell lines, which in turn may explain the difference in CASCADE 3.0 model performance. In addition to Colo205, other well-performing cell lines include the gastric and prostate cancer cell lines. Interestingly, Colo205, AGS and DU145 all originate from the same tissue type, the epithelial, hinting to a pattern in the model’s performance. By grouping the cell lines by their tissues of origin, it became evident that the model had a tendency to perform considerably better for the epithelial cancers (i.e., adenocarcinomas and carcinomas), and not only for the colorectal adenocarcinoma that it was specified for. Tumors are often classified by the organ they arise in. However, the molecular profiling of major cancer types has revealed surprising similarities between the molecular profiles of tumors arising from the same tissue type, but in different organs (Lin et al., 2017). For instance, the oncogenic role of the newly added Hippo, Hedgehog, and Notch modules is well reported in both prostate cancer (Zhang et al., 2015; Su and Xin, 2016; Buttyan et al., 2018) and gastric cancer (Kang et al., 2016; Yao et al., 2017; Akyala and Peppelenbosch, 2018). Together with the notion that targeted therapy based on molecular features is more effective (Senft et al., 2017), as practiced in precision medicine, the observation that CASCADE 3.0 has an overall better performance on cell lines displaying similar molecular phenotypes, may provide a handle on further optimizing logical modules for cancer cell line sets with shared other molecular profiles. This hypothesis could be further investigated in larger scale datasets, where the predictions of the model can be tested against additional drug combination data, as for example the drug synergy data reported in DrugComb (Zagidullin et al., 2019) and SYNERGxDB (Seo et al., 2020), and potentially in a broader set of cell lines.

The combination of proteomics with genomics data has been proposed as the most effective way to infer the state of an entity (Senft et al., 2017), corroborating our observation that models trained to the combined data set tend to perform generally better. The noticeable variation of the performance with different training data even for a specific cell line underlines the importance of correctly assessing the entities’ states before training a model, which would need careful, high-quality assays for all proteins represented in the logical model. In most biological systems, however, it is assumed that the state of only a specific subset of its nodes is rather sufficient to control the global state of the system (Gao et al., 2014; Dnyane et al., 2018; Yang J. M. et al., 2018). Based on this, the accurate identification of the states of a well-chosen subset of nodes in the model rather than the majority of its nodes can be an attractive alternative (Niederdorfer et al., 2020). However, since the behavior of Boolean networks depends on multiple node and network features (Kauffman et al., 2004; Kochi et al., 2014), and often on the combined effect of those individual features (Kochi et al., 2014), it is essential to identify which of those features can be best used to assess the importance of a node for the global state of a system. Several features, including well-established or novel topology metrics (e.g., in-degree, out-degree, various path lengths, and centrality measures) and dynamical characteristics (e.g., bias and sensitivity of Boolean functions, presence of feedback loops), have been proposed to identify those nodes (Kochi et al., 2014; Sheikhahmadi et al., 2015; Wang et al., 2017). These findings suggest that further work on identifying such ‘high leverage’ nodes, or even complete modules that are critical for a model’s performance and whose state therefore should be accurately assessed, is much needed.

Most of the observed synergies that could be predicted involve one of the PI3K/AKT, JNK/p38, or RAC modules. These modules play a central regulatory role in both normal and malignant cells, and many studies have already investigated and supported the effectiveness of combinatorial over single-agent treatment targeting these pathways, either in combination with each other or together with other pathways (Jain et al., 2017; Pons-Tostivint et al., 2017; Rocca et al., 2018). Alternatively, the apparent higher success rate for these modules may also be a consequence of the bias of this study toward drugs targeting the PI3K/AKT and JNK/p38 modules (seven of the 18 drugs). The classification of the modules (see Figure 3) based on whether they promote apoptosis, metastasis or proliferation, when the pathway they represent is active, revealed that the model fails to predict synergies for drugs targeting module combinations from different functional classes (apoptosis and proliferation), while it could predict most synergies that involved a combinatorial targeting of proliferation-associated modules. This observation may indicate a lack of regulatory detail in specific subparts, namely the apoptosis-related modules, or their cross-talk with the other parts of the network, especially given their direct interaction with the *Antisurvival* phenotype. To test this, additional curation efforts could be performed in an iterative way while testing model performance. Additional reasons that might affect the performance of the model in drug synergy prediction may be found in the lack of knowledge about the specificity of some cancer drugs (Rázga and Némethová, 2017). They may have unforeseen off-target effects that for a variety of reasons cannot be taken into account in the perturbed model simulations, which could seriously affect the model’s performance (Saginc et al., 2017). For the moment, there are additional frontiers that need to be crossed before logical model-based therapy design can become relevant for the clinic.

In summary, this paper illustrates that middle-out model building provides an efficient approach to extend and optimize a logical model for specific cancer cell lines, or even individual patients, for more accurate drug effect simulations. The results illustrate that guided extensions of models to optimize their representation of a disease system can provide important insights and guide experimental design toward the identification of effective drug combinations. This approach allows the prioritization of the proposed synergies in a pre-clinical setting, to facilitate the selection of candidate drugs combinations that should be experimentally tested on cell lines.

## Data Availability Statement

The datasets generated for this study are available on request to the corresponding author.

## Author Contributions

ET and MK designed the approach. MK supervised the project. ET carried out the multi-omics data analysis and model extensions and analyses. VT provided help with the logical model simulations. BN made available and provided guidance in the use of the CASCADE 2.0 model for model extensions. ÅF and MV constructed and made available the NTNU logical model simulation pipeline. All authors contributed to the manuscript.

## Conflict of Interest

The authors declare that the research was conducted in the absence of any commercial or financial relationships that could be construed as a potential conflict of interest.

## Footnotes

1 https://druglogics.github.io/druglogics-doc/index.html
