# Supplementary material for: A Middle-Out Modeling Strategy to Extend a Colon Cancer Logical Model Improves Drug Synergy Predictions in Epithelial-Derived Cancer Cell Lines
Source: Front Mol Biosci. 2020 Oct 9;7:502573. doi: 10.3389/fmolb.2020.502573 (PMC7581946; doi:10.3389/fmolb.2020.502573)
Supplement: Supplementary file 1 [file Data_Sheet_1.pdf]

Supplementary Material to the paper “A middle-out modeling strategy to extend a colon cancer logical model improves drug synergy predictions in epithelial-derived cancer cell lines.”

In this document, we present the information referred to in the main document as Supplementary Material.

Content:

Tools and databases **1**

Patient classification with CMS data **2**

Functional analysis by enrichment analysis **4**

Training data size reduction **9**

Tools and databases

Table S1: Summary of the tools, software, databases and their version numbers used in this project.

Purpose of use	Tool / Database	Version	Link
Omics data analysis & Network visualization	R	3.5.1	https://www.r-project.org/
	R Studio	1.1.456	https://www.rstudio.com/
	Bioconductor	3.7 - 3.8	https://bioconductor.org/
	GISTIC 2.0	6.14	http://software.broadinstitute.org/cancer/software/genepattern/modules/docs/GISTIC_2.0
	MutSigCV	1.2	http://software.broadinstitute.org/cancer/software/genepattern/modules/docs/MutSigCV
	Gene Ontology (GO) terms	2018-Apr-4	http://geneontology.org/
	MSigDB	6.3	http://software.broadinstitute.org/gsea/msigdb
	COSMIC	87	https://cancer.sanger.ac.uk/cosmic

	OpenTargets	3.11.4	https://www.opentargets.org/
	Cytoscape	3.7	https://cytoscape.org/
Model expansion	Signor	2.0	https://signor.uniroma2.it/
	Reactome	66-67	https://reactome.org/
	KEGG (Kyoto Encyclopedia of Genes and Genomes)	87.0 - 89.1	https://www.genome.jp/kegg/
	ACSN (Atlas of Cancer Signaling Networks)	2.0	https://acsn.curie.fr/index_old.html

Patient classification with CMS data

To classify the available omics data to the Consensus Molecular Subtypes we performed a gene expression-based classification of TCGA-COAD patients according to the Consensus Molecular Subtypes (CMS) of colorectal cancer (Guinney et al. 2015), using the CMScaller R package (Eide et al. 2017). The classification was based on the nearest distance between a sample's normalized and log2-transformed gene expression data and cancer-cell, coherently upregulated gene expression templates enriched in each CRC subtype. Prediction confidence was estimated by a series of permutation tests and corrected for multiple testing. A threshold of FDR < 0.05 for the patient assignment was used. RNA sequencing data were available for 478 tumor samples of 472 unique patients. CMScaller classified 427 patients in one of the four CMSs with FDR < 0.05. The remaining 45 samples could not be assigned to a subtype with sufficient confidence and are assumed to represent transitioning phenotypes, rather than outliers or an additional subtype, as in the original publication of the CMS [23]. The representation of each subtype was similar to the percentages found in the original CMS publication, except for CMS4, which was found to be overrepresented in the COAD cohort. The results of the classification are presented in Table S2.

Table S2: Number of patients of the TCGA-COAD cohort, classified for the different consensus molecular subtypes of colorectal cancer using the CMScaller package.

**Subtype**	**No. of patients**	**Percentage of total samples**
CMS1	82	17 %
CMS2	139	29.5 %
CMS3	70	15 %
CMS4	136	29 %
Not assigned	45	9.5 %

After the classification of patients to CMS groups, the individual omics data sets were used to identify those genes that were affected in their sequence or at their transcription level in each subtype.

The affected genes were next used to perform an overrepresentation analysis, the results of which can be seen in Figures S1, S2, and S3.

Functional analysis by enrichment analysis

Genes that were affected by mutations or in their gene expression (or both) for each of the four CMS classes (CMS1, CMS2, CMS3, and CMS4) were tested against the KEGG database (Kyoto Encyclopedia of Genes and Genomes), ACSN (Atlas of Cancer Signaling Networks), and Reactome, to find their overrepresentation in cancer-related pathways.

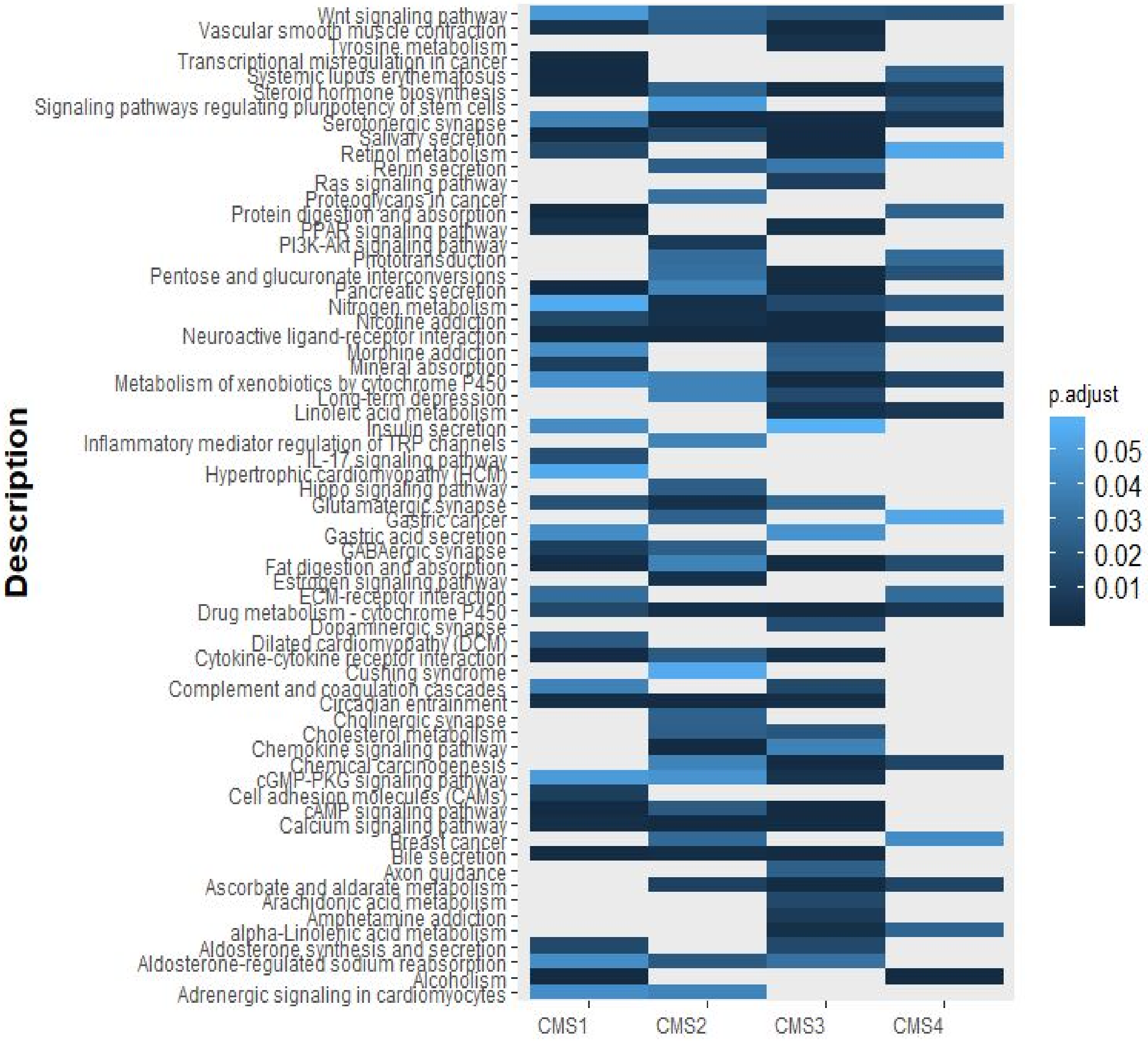

Figure S1: Enriched KEGG pathways. A heat-map indicates KEGG pathways enriched in affected genes, for each of the Consensus Molecular Subtypes, indicated below the graph, identified by the clusterProfiler package. P-values were corrected for multiple testing with the Benjamini-Hochberg method.

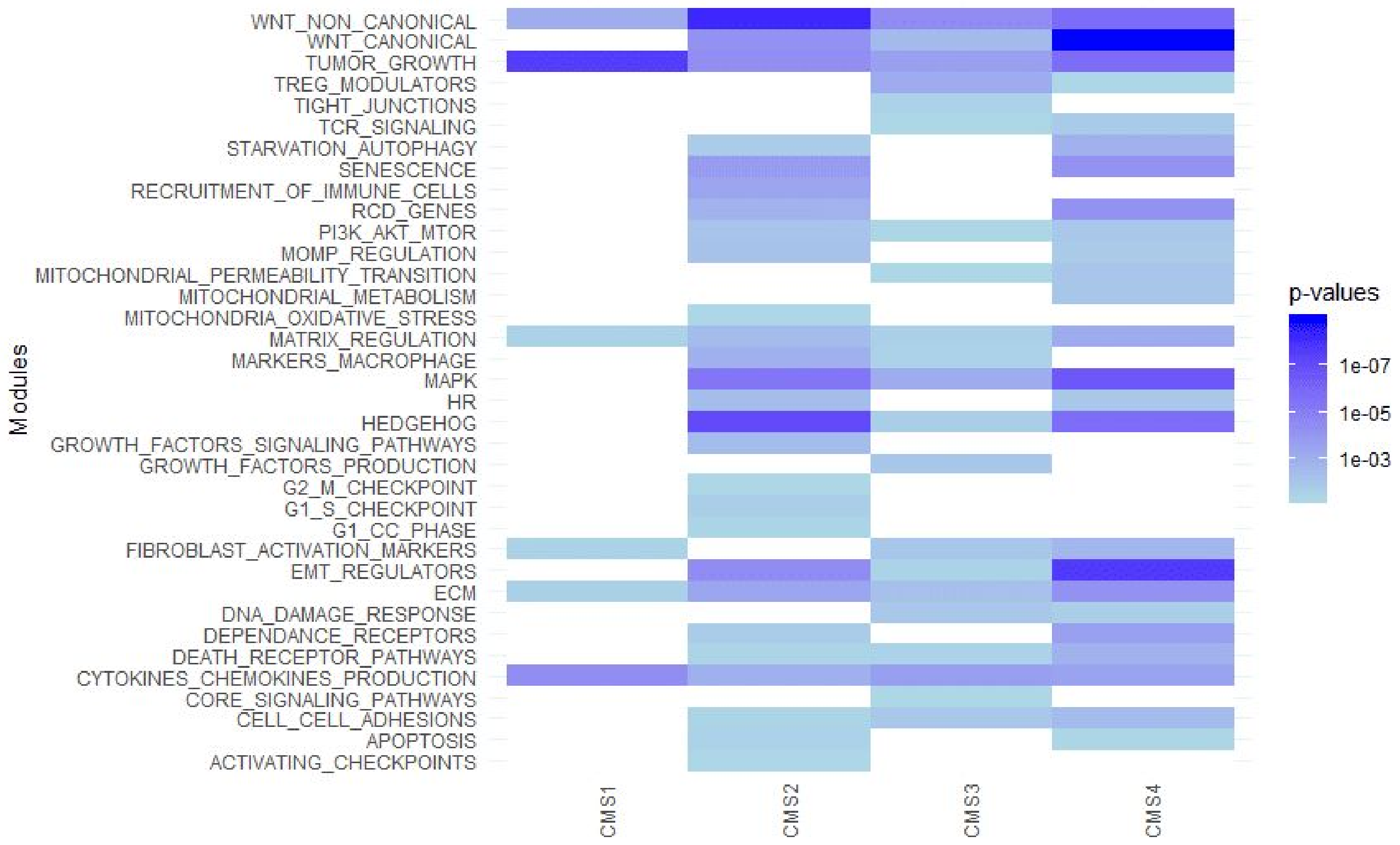

Figure S2: Enriched ACSN pathways. The heatmap shows modules with overrepresented genes for each of the Consensus Molecular Subtypes, identified by the ACSNMineR package. P-values were corrected for multiple testing with the Benjamini-Hochberg method.

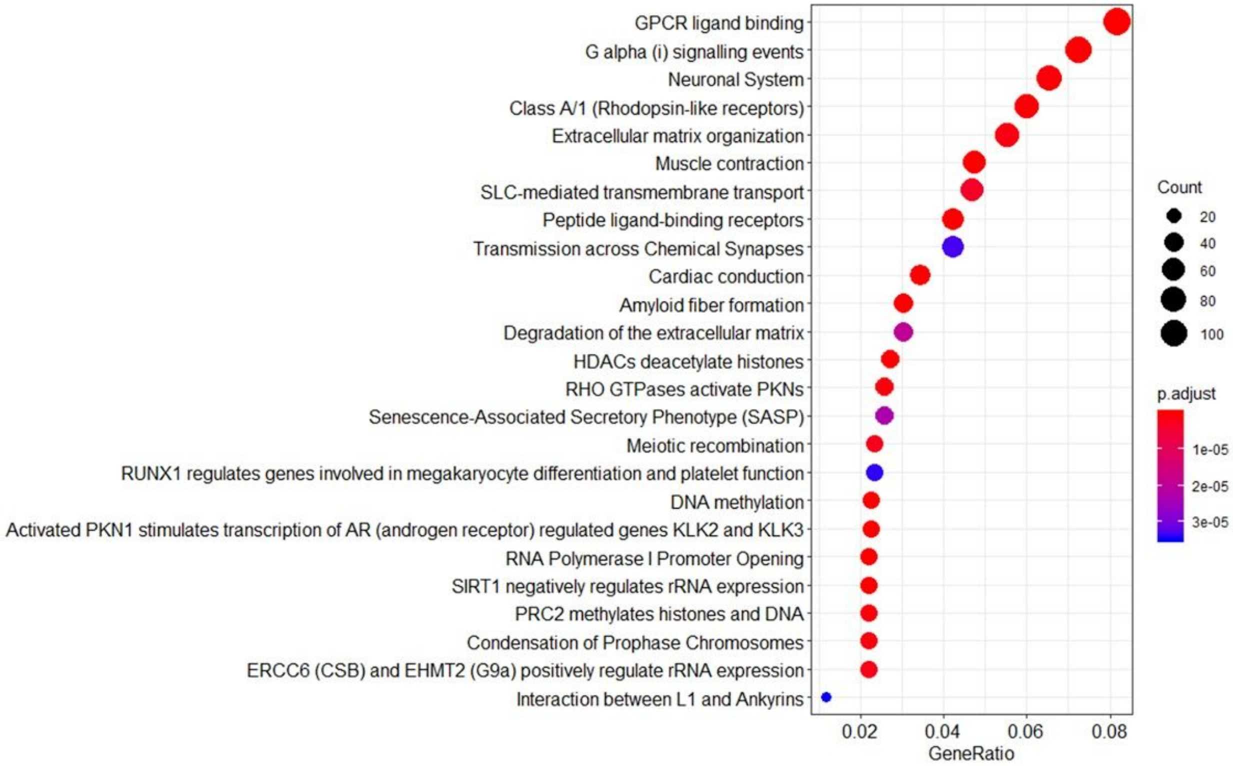

GPCR ligand binding
G alpha (i) signalling events
Neuronal System
Class A/1 (Rhodopsin-like receptors)
Extracellular matrix organization
Muscle contraction
SLC-mediated transmembrane transport
Peptide ligand-binding receptors
Transmission across Chemical Synapses
Cardiac conduction
Amyloid fiber formation
Degradation of the extracellular matrix
HDACs deacetylate histones
RHO GTPases activate PKNs
Senescence-Associated Secretory Phenotype (SASP)
Meiotic recombination
RUNX1 regulates genes involved in megakaryocyte differentiation and platelet function
DNA methylation
Activated PKN1 stimulates transcription of AR (androgen receptor) regulated genes KLK2 and KLK3
RNA Polymerase I Promoter Opening
SIRT1 negatively regulates rRNA expression
PRC2 methylates histones and DNA
Condensation of Prophase Chromosomes
ERCC6 (CSB) and EHMT2 (G9a) positively regulate rRNA expression
Interaction between L1 and Ankyrins
0.02
0.04
0.06
0.08
GeneRatio
Count
20
40
60
80
100
p.adjust
1e-05
2e-05
3e-05

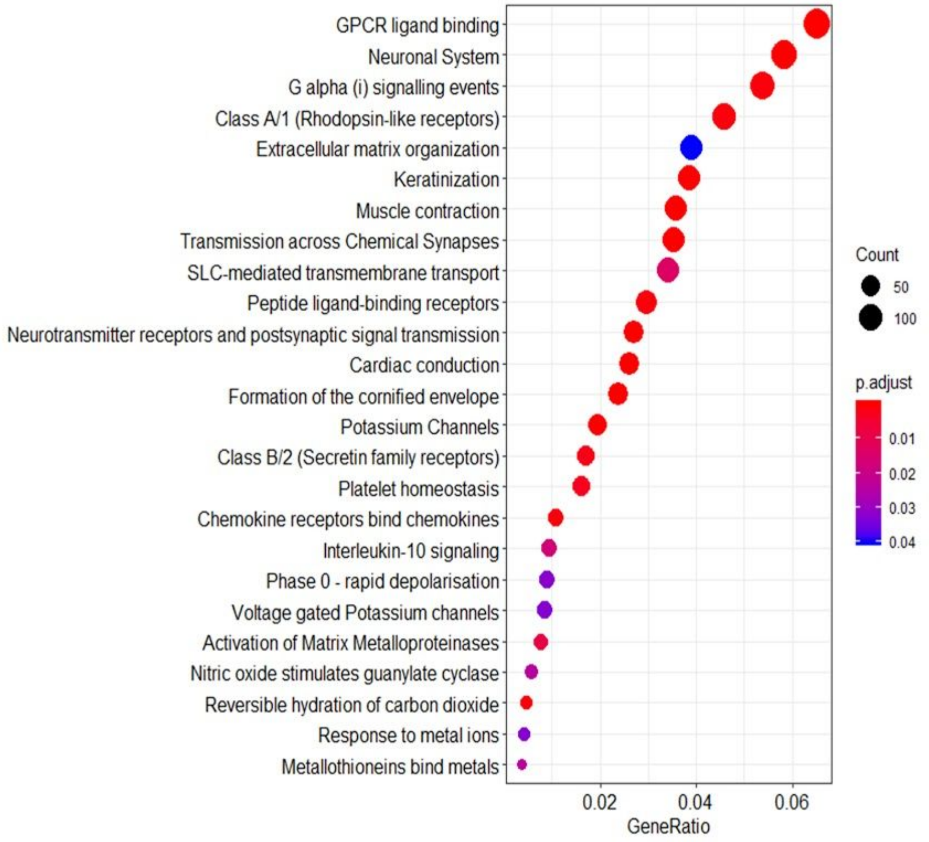

GPCR ligand binding
Neuronal System
G alpha (i) signalling events
Class A/1 (Rhodopsin-like receptors)
Extracellular matrix organization
Keratinization
Muscle contraction
Transmission across Chemical Synapses
SLC-mediated transmembrane transport
Peptide ligand-binding receptors
Neurotransmitter receptors and postsynaptic signal transmission
Cardiac conduction
Formation of the cornified envelope
Potassium Channels
Class B/2 (Secretin family receptors)
Platelet homeostasis
Chemokine receptors bind chemokines
Interleukin-10 signaling
Phase 0 - rapid depolarisation
Voltage gated Potassium channels
Activation of Matrix Metalloproteinases
Nitric oxide stimulates guanylate cyclase
Reversible hydration of carbon dioxide
Response to metal ions
Metallothioneins bind metals
0.02
0.04
0.06
GeneRatio
Count
50
100
p.adjust
0.01
0.02
0.03
0.04

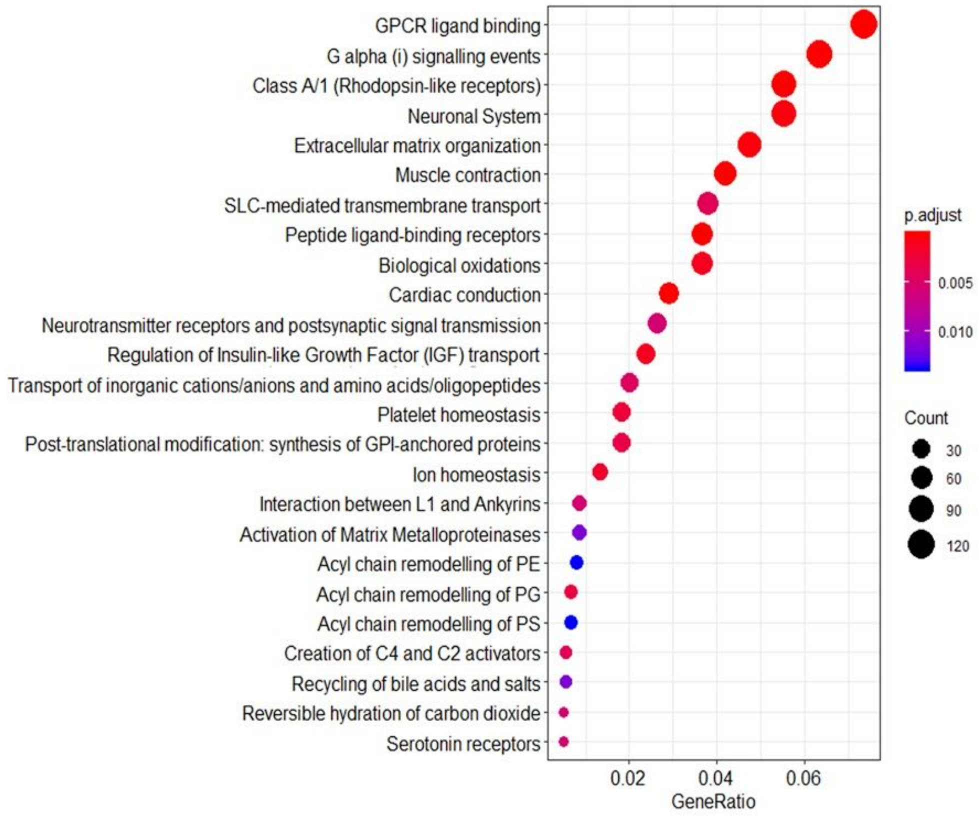
GPCR ligand binding
G alpha (i) signalling events
Class A/1 (Rhodopsin-like receptors)
Neuronal System
Extracellular matrix organization
Muscle contraction
SLC-mediated transmembrane transport
Peptide ligand-binding receptors
Biological oxidations
Cardiac conduction
Neurotransmitter receptors and postsynaptic signal transmission
Regulation of Insulin-like Growth Factor (IGF) transport
Transport of inorganic cations/anions and amino acids/oligopeptides
Platelet homeostasis
Post-translational modification: synthesis of GPI-anchored proteins
Ion homeostasis
Interaction between L1 and Ankyrins
Activation of Matrix Metalloproteinases
Acyl chain remodelling of PE
Acyl chain remodelling of PG
Acyl chain remodelling of PS
Creation of C4 and C2 activators
Recycling of bile acids and salts
Reversible hydration of carbon dioxide
Serotonin receptors
0.02
0.04
0.06
GeneRatio
p.adjust
0.005
0.010
Count
30
60
90
120

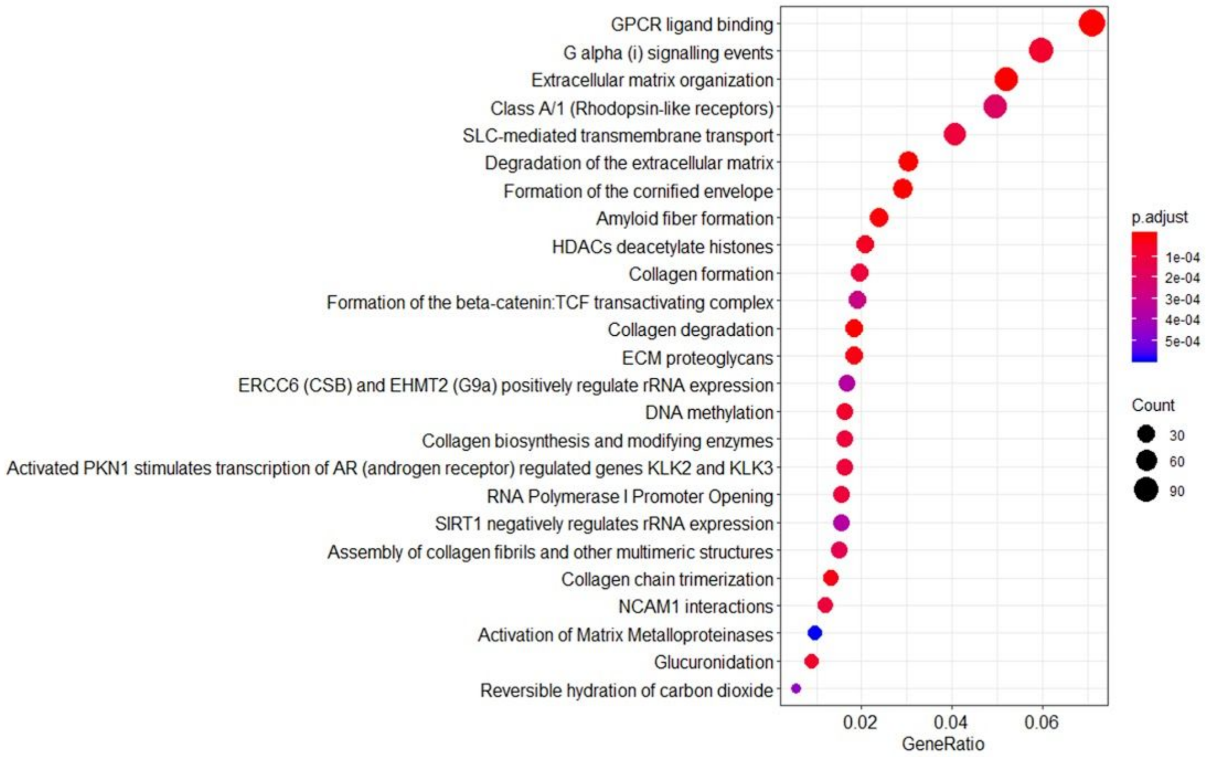
GPCR ligand binding
G alpha (i) signalling events
Extracellular matrix organization
Class A/1 (Rhodopsin-like receptors)
SLC-mediated transmembrane transport
Degradation of the extracellular matrix
Formation of the cornified envelope
Amyloid fiber formation
HDACs deacetylate histones
Collagen formation
Formation of the beta-catenin:TCF transactivating complex
Collagen degradation
ECM proteoglycans
ERCC6 (CSB) and EHMT2 (G9a) positively regulate rRNA expression
DNA methylation
Collagen biosynthesis and modifying enzymes
Activated PKN1 stimulates transcription of AR (androgen receptor) regulated genes KLK2 and KLK3
RNA Polymerase I Promoter Opening
SIRT1 negatively regulates rRNA expression
Assembly of collagen fibrils and other multimeric structures
Collagen chain trimerization
NCAM1 interactions
Activation of Matrix Metalloproteinases
Glucuronidation
Reversible hydration of carbon dioxide
0.02
0.04
0.06
GeneRatio
p.adjust
1e-04
2e-04
3e-04
4e-04
5e-04
Count
30
60
90

Figure S3: Enriched Reactome maps. The plots show pathways significantly enriched for genes from CMS1 (S3(A)), CMS2 (S3(B)), CMS3 (S3(C)) and CMS4 (S3(D)) as identified by the clusterProfiler package. P-values were corrected for multiple testing with the Benjamini-Hochberg method and a threshold of 0.05 was used.

Training data size reduction

Initially, the drug synergy prediction analysis was performed using four different training data sets presented in table S3.

Table S3: Description of the training data used for calibrating the CASCADE 3.0 model to each of the eight cancer cell lines, prior to logical model simulations and drug synergy prediction.

Name	Data used	Total no. of inferred states	States for CASCADE 3.0 entities	States for CASCADE 3.0 nodes
**Combination based**	Copy number variation, Gene expression, RPPA for total protein abundance, RPPA for phosphosites	757	295	182
**mRNA expression based**	Gene expression	331	119	81
**Drug Screen based**	Data from MD Anderson Cell Line Project (MCLP)	79	37	30
**TF-activity based**	Data from "Genomics of Drug Sensitivity in Cancer" (GDSC) project	26	21	15

The training data sets with node activity states inferred from genome-wide omics data are so comprehensive that they specify states for almost all of the model's nodes. This is not necessarily a good thing, as we have observed with various models (CASCADE 1.0, 2.0 and 3.0) that fitting them to a predicted state for almost all of their nodes tends to decrease their prediction accuracy, relative to models that are trained to only a smaller set of predicted states. Because of that observation, we reduced the combination based training data to only those entities whose state was predicted in at least one of the smaller training data sets. Together, these small data sets contain inferred activity states for 123 entities, represented as 85 nodes of the network (some entities belong to the same family node). After reducing the combination and mRNA expression-based training data to only those 85 nodes, the model presented a considerable improvement in its performance.
